# Quantitative Ultrasonographic and Shear Wave Elastographic Assessment of Infraorbital Tissue Changes After Non-cross-linked Hyaluronic Acid and Amino Acid Therapy

**DOI:** 10.7759/cureus.109369

**Published:** 2026-05-21

**Authors:** Zekai Kutlubay, Miraç Zengin, Aykan Kargın, Vefa Salt, Rauf Hamid

**Affiliations:** 1 Dermatology, Istanbul University School of Medicine, Ankara, TUR; 2 Dermatology, Şehit Prof. Dr. İlhan Varank Sancaktepe Eğitim ve Araştırma Hastanesi, İstanbul, TUR; 3 Radiology, Istanbul Physical Therapy and Rehabilitation Training and Research Hospital, Istanbul, TUR; 4 Radiology, Cerrahpasa Faculty of Medicine, Istanbul, TUR; 5 Radiology, T.C. Sağlık Bakanlığı Sungurlu Devlet Hastanesi, Çorum, TUR

**Keywords:** amino acid therapy, elastography, infraorbital rejuvenation, lower eyelid, non-cross-linked hyaluronic acid

## Abstract

Objective

The aim of this prospective single-arm interventional study was to quantitatively assess changes in infraorbital tissue thickness and elasticity after non-cross-linked hyaluronic acid and amino acid therapy using B-mode ultrasonography and two-dimensional shear wave elastography (2D-SWE).

Methods

This prospective, open-label, single-arm interventional study was conducted between January 2024 and December 2024 and included 40 female patients undergoing non-cross-linked hyaluronic acid and amino acid therapy for infraorbital rejuvenation. Treatment-related changes in infraorbital tissue thickness and elasticity were evaluated using B-mode ultrasonography and 2D-SWE before treatment and three months after the initial treatment. Patient-reported satisfaction, physician-assessed clinical response, and short-term safety outcomes were also assessed.

Results

In the patient satisfaction assessment, 34 patients (85%) were satisfied with the procedure, whereas six patients (15%) were not satisfied. Lower eyelid epidermis-dermis thickness increased from 1.34 ± 0.34 mm before treatment to 2.05 ± 0.39 mm after treatment, corresponding to an approximately 53.0% increase (p<0.001). Palpebral orbicularis oculi thickness increased from 0.95 ± 0.29 mm to 1.33 ± 0.41 mm, corresponding to an approximately 40.0% increase (p<0.001). Lower eyelid epidermis-dermis elasticity increased from 3.96 ± 0.95 kPa to 4.99 ± 1.28 kPa, corresponding to an approximately 26.0% increase (p<0.001), and palpebral orbicularis oculi elasticity increased from 4.39 ± 1.07 kPa to 5.36 ± 1.75 kPa, corresponding to an approximately 22.1% increase (p<0.001). Retro-orbicularis oculi fat (ROOF) pad elasticity increased from 4.44 ± 1.42 kPa to 5.01 ± 1.77 kPa, corresponding to an approximately 12.8% increase; however, this change did not reach statistical significance (p=0.068).

Conclusion

Two sessions of non-cross-linked hyaluronic acid and amino acid therapy were associated with measurable short-term increases in infraorbital epidermis-dermis thickness, palpebral orbicularis oculi thickness, and selected tissue elasticity parameters on B-mode ultrasonography and 2D-SWE, with no serious adverse events observed during the three-month follow-up period.

## Introduction

The periorbital region plays a central role in facial aesthetics because it strongly influences the perception of youthfulness, vitality, and overall facial appearance [1]. Consequently, aesthetic concerns in this area are common, and various treatment approaches have been used to improve infraorbital skin quality and signs of aging [2].

The skin surrounding the eyes is thin and dry, with the eyelid skin measuring 0.5 to 1 mm thick. This area is characterized by a thin epidermis and dermis. A distinctive feature of the infraorbital region is the lack of a fat layer supporting the skin surface, which is considered a significant contributing factor to the early signs of aging in this area [2]. Factors such as repeated facial expressions, exposure to ultraviolet radiation, and smoking can contribute to decreased skin elasticity and the development of signs of aging.

Like other areas of the face, the infraorbital region is characterized by signs of aging, such as fine and deep wrinkles, pouches, dryness, and changes in pigmentation. Several non-surgical approaches have been used for infraorbital rejuvenation, including topical treatments, mechanical and chemical resurfacing procedures, laser- and radiofrequency-based devices, fillers, botulinum toxin, mesotherapy, and platelet-rich plasma therapy [3,4].

The extracellular matrix is a complex and dynamic network in all body tissues. This network surrounds cells, provides structural and mechanical support, and mediates various important biological processes to support tissue formation and function. The main structural contributors to the extracellular matrix are the fibrous proteins collagen and elastin. Collagen is the major component of the extracellular matrix. Skin thinning (as part of aging) is related to different factors (exposure to sunlight, smoking, and chronological aging), which also lead to collagen deficiency. Collagen is characterized by Gly-X-Y amino acid triplet repeats, where Gly represents glycine, and proline and hydroxyproline are frequently located at positions X and Y, respectively. However, amino acids such as leucine or lysine can also be located at the Y position [5].

Non-cross-linked hyaluronic acid combined with amino acids has been proposed as a regenerative injectable approach that may support dermal hydration, extracellular matrix remodeling, and skin quality improvement [6-8]. However, the biological mechanisms underlying these effects remain incompletely defined, and objective imaging-based data regarding tissue-level changes after infraorbital application are limited.

Although non-cross-linked hyaluronic acid and amino acid-based treatments are increasingly used for periorbital rejuvenation, most previous evaluations have relied mainly on clinical assessment and patient-reported outcomes. Objective imaging-based data on treatment-related changes in infraorbital tissue thickness and biomechanical properties remain limited. Therefore, this study aimed to quantitatively assess changes in infraorbital tissue thickness and elasticity after non-cross-linked hyaluronic acid and amino acid therapy using B-mode ultrasonography and two-dimensional shear wave elastography (2D-SWE). The secondary objectives were to evaluate patient-reported satisfaction, physician-assessed clinical response, and short-term safety outcomes.

## Materials and methods

Materials

A commercially available injectable formulation containing non-cross-linked hyaluronic acid and amino acids (Jalupro® Young Eye; Professional Derma SA, Lugano, Switzerland) was used in this study. The product is CE Class III certified under Medical Device Directive 93/42/EEC. The 1 mL pre-filled syringe contains the following components: 12 mg of non-cross-linked hyaluronic acid (8 mg low molecular weight and 4 mg medium molecular weight), along with a combination of seven amino acids (glycine, L-proline, L-lysine, L-leucine, L-alanine, valine, and arginine) and the EyeSeryl (acetyl tetrapeptide-5) complex.

Methods

This prospective, open-label, single-arm interventional study was conducted between January 2024 and December 2024 and included 40 female patients undergoing non-cross-linked hyaluronic acid and amino acid therapy for infraorbital rejuvenation. The primary objective was to evaluate treatment-related changes in infraorbital tissue thickness and elasticity using B-mode ultrasonography and 2D-SWE. Secondary outcomes included patient-reported satisfaction, physician-assessed clinical response, and short-term safety outcomes. A within-subject design was used, in which each patient served as her own control by comparing baseline and post-treatment measurements. Paired statistical tests were used to compare pre- and post-treatment values.

Eligible participants were female patients aged over 18 years who presented with infraorbital aesthetic concerns, including puffiness, hyperpigmentation, wrinkles, or impaired skin quality. Patients were required to have no history of surgical or cosmetic procedures in the infraorbital region within the previous six months. The inclusion of only female participants was intended to improve internal homogeneity; however, this sex-restricted sampling strategy limits the generalizability of the findings to male patients and mixed-sex populations.

The exclusion criteria included systemic conditions such as sepsis, immunosuppression, malignancy, pregnancy, or breastfeeding. Individuals who had undergone any surgical, cosmetic, or active dermatologic treatment affecting the infraorbital region within the previous six months were also excluded. Patients with active periorbital dermatitis, infection, or inflammatory skin disease at the time of treatment were not included. Medication history was reviewed before treatment, and patients using medications that could increase injection-related risks, such as anticoagulant or antiplatelet therapy, were evaluated individually before inclusion. Furthermore, male patients and individuals under the age of 18 were not included.

All participants provided written informed consent prior to study inclusion. The study protocol was conducted in accordance with the Declaration of Helsinki and Good Clinical Practice guidelines and was approved by the Istanbul University-Cerrahpaşa, Cerrahpaşa Faculty of Medicine Clinical Research Ethics Committee, Istanbul, Turkey (Approval No: E-83045809-604.01.01-854220).

Intervention protocol

All participants received a commercially available injectable formulation containing non-cross-linked hyaluronic acid and a defined amino acid complex (Jalupro® Young Eye). The treatment protocol consisted of two sessions administered at three-week intervals. Each session involved the administration of 1 mL of the solution, with 0.5 mL injected into each infraorbital region. The injection plane was standardized as the deep dermis and was determined according to visible micropapule formation, minimal resistance during injection, and avoidance of bolus placement in deeper tissues. The product was delivered as multiple small micropapules rather than as a bolus injection. Injections were performed using a 13 mm, 32-gauge needle, employing a standardized micropapular technique at approximately 1 cm intervals. All injections were performed by the same experienced physician trained in infraorbital injectable procedures, using the same bilateral injection scheme at both treatment sessions. No additional infraorbital cosmetic procedure was performed during the three-month follow-up period. Standardized digital photographs were obtained before the procedure and three months after the initial treatment for comparative evaluation.

Clinical assessment

Clinical outcomes were evaluated using both patient-reported measures and physician-based assessment tools. Patient-reported outcomes included perceived changes in skin dryness, wrinkles, hyperpigmentation, and puffiness in the periorbital area. Physician assessment was performed using a 10-point clinical response scale categorized as follows: 0-2 (no response), 3-4 (slight response), 5-6 (moderate response), 7-8 (good response), and 9-10 (very good response). Overall patient satisfaction was recorded three months after the initial treatment using a structured questionnaire developed by the authors for this study. Validated aesthetic outcome scales, such as the Global Aesthetic Improvement Scale (GAIS), were not used. Outcome assessments were not performed in a blinded manner because of the open-label, single-arm study design. Therefore, the clinical response scale and questionnaire were interpreted as supportive subjective outcomes rather than primary efficacy endpoints; the primary evaluation was based on quantitative B-mode ultrasonographic and 2D-SWE measurements.

Ultrasonographic evaluation

Objective assessment was performed using ultrasonographic imaging at baseline (pre-treatment) and three months after the initial intervention. The evaluation focused on key anatomical structures within the infraorbital region, including the epidermis-dermis layer, the thickness of the palpebral portion of the orbicularis oculi muscle, and the retro-orbicularis oculi fat (ROOF) pad.

All examinations were conducted using B-mode ultrasonography and 2D-SWE with a Canon Aplio i800 ultrasound system (Canon Medical Systems Inc., Tokyo, Japan) equipped with a 12-16 MHz linear transducer. Epidermis-dermis thickness and the thickness of the palpebral portion of the orbicularis oculi muscle were measured using B-mode ultrasonography.

A standardized imaging protocol was applied to minimize measurement variability. A generous amount of coupling gel was used to create a cushion between the transducer and the skin, thereby avoiding compression artifacts, and care was taken not to exert pressure on the examined tissues during image acquisition. If image quality was insufficient, measurements were repeated until stable and artifact-free images were obtained. All ultrasonographic and elastographic measurements were performed according to the same standardized imaging protocol by an operator experienced in high-frequency ultrasonography and 2D-SWE of superficial soft tissues. To reduce measurement variability, three repeated measurements were obtained from each anatomical region, and the mean value was used for statistical analysis. Formal intra-observer and inter-observer variability analyses were not performed.

**Figure 1:**
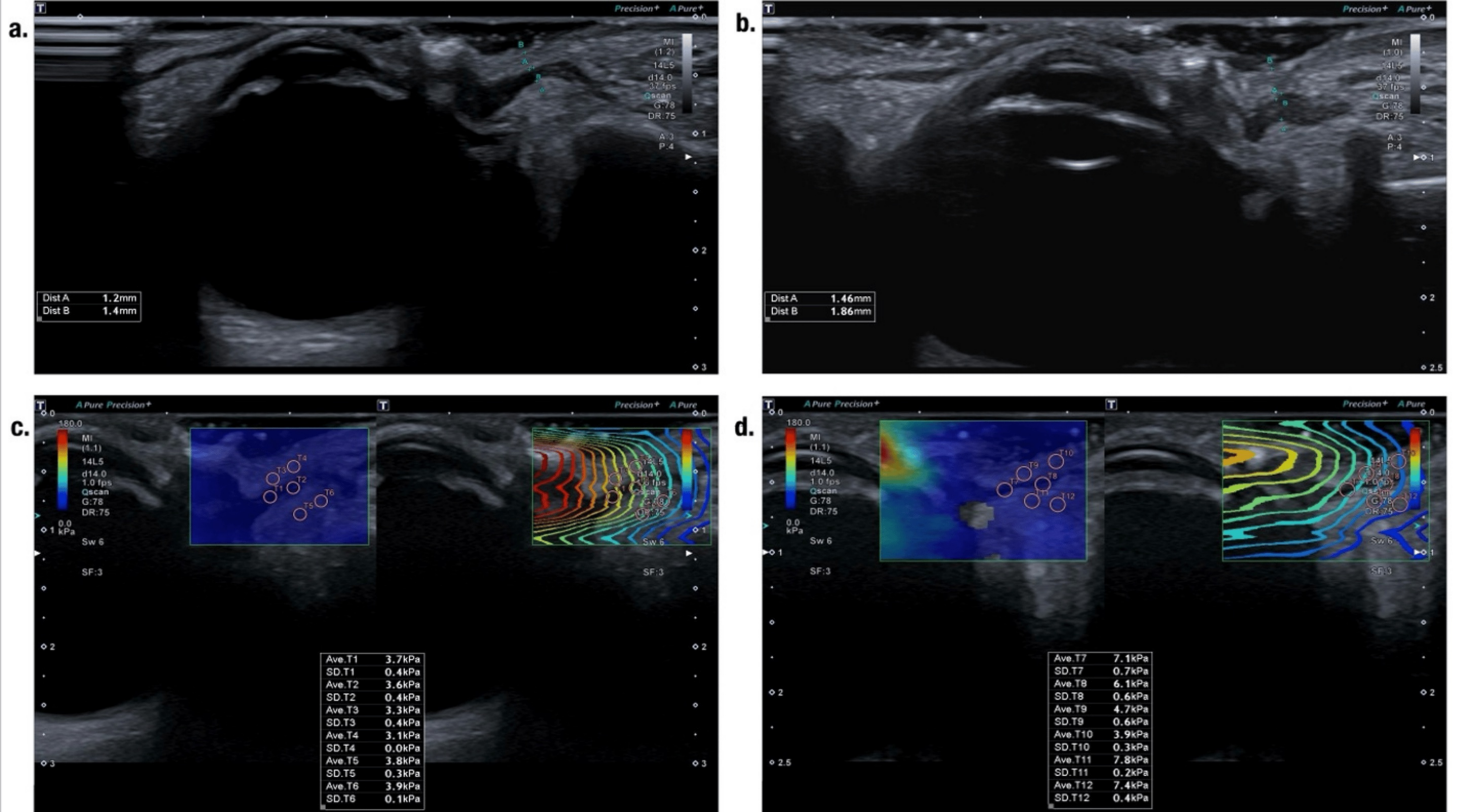
Representative ultrasonographic and elastographic findings before and after infraorbital rejuvenation treatment Ultrasonographic images of the lower eyelid of a representative patient before (a, c) and after (b, d) the procedure. Upper row (a, b): Gray-scale images show the comparison of the epidermis-dermis thickness and orbicularis oculi palpebral part thickness pre- and post-procedure. The epidermis-dermis thickness of the lower eyelid increased from 1.2 mm to 1.46 mm, while the thickness of the palpebral part of the orbicularis oculi increased from 1.4 mm to 1.86 mm after the procedure. Lower row (c, d): Elastography measurements indicate increased mean elasticity values (E-mean) after the procedure for the epidermis-dermis layer (from 3.2 kPa to 4.3 kPa), the palpebral part of orbicularis oculi (from 3.7 kPa to 6.6 kPa), and the retro-orbicularis oculi fat pad layer (from 3.9 kPa to 7.6 kPa).

Among elastographic techniques, 2D-SWE was preferred due to its higher reproducibility and lower operator dependency. E-mean values were obtained by placing a circular region of interest (ROI) measuring 1 × 1 mm in each evaluated anatomical region. This small ROI size was selected because the infraorbital anatomical layers are thin and closely adjacent to each other; therefore, a larger ROI could have included neighboring tissues and increased partial-volume-related measurement variability. ROI placement was performed on artifact-free images within the visually defined target layer, avoiding tissue interfaces whenever possible. Quantitative elasticity values were calculated as the mean of three separate measurements and expressed in kPa (Figure 1).

Study design considerations

A within-subject analytical design was employed, in which each participant served as their own control. Pre-treatment measurements were directly compared with post-treatment outcomes in order to evaluate treatment-related changes. This design was selected because the primary objective was to quantify short-term intraindividual changes in predefined ultrasonographic and elastographic parameters after treatment. However, the single-arm, open-label nature of the study should be considered when interpreting the results, as the absence of a parallel control group prevents complete exclusion of potential confounding factors, including time-related changes, placebo effects, or expectation-related influences. Therefore, the within-subject comparisons were interpreted as preliminary intraindividual changes and not as a substitute for a parallel control group; a controlled comparative design would be required to confirm causality and to more reliably distinguish treatment-related effects from nonspecific or time-dependent changes.

Statistical analysis

Descriptive statistics were presented as mean ± standard deviation, median, minimum, and maximum values, as appropriate. The normality of data distribution was assessed using the Kolmogorov-Smirnov test, and non-parametric tests were applied accordingly. The primary statistical analysis focused on within-subject pre- and post-treatment comparisons of ultrasonographic and elastographic parameters, which were performed using the Wilcoxon signed-rank test. Independent-group tests were not used to evaluate the primary treatment effect. The Mann-Whitney U test was used only for exploratory comparisons between independent subgroups, such as satisfied versus non-satisfied patients, and categorical variables were analyzed using the chi-square test. When treatment-response categories were compared, these analyses were considered exploratory secondary analyses and were interpreted descriptively in the context of the single-arm study design. Comparisons of continuous variables across more than two treatment-response categories were performed using the Kruskal-Wallis test. Categorical variables were compared using the chi-square test or Fisher’s exact test, as appropriate, according to expected cell counts. Because of the exploratory nature of the study, formal confidence intervals and effect size estimates were not calculated. Percentage changes were reported to facilitate the interpretation of the magnitude of pre- and post-treatment differences. No formal correction for multiple comparisons was applied; therefore, p-values from secondary subgroup and treatment-response analyses were interpreted descriptively and should be considered hypothesis-generating. Correlation analyses were conducted using Spearman’s rank correlation coefficient. All statistical analyses were performed using IBM SPSS Statistics for Windows, Version 28.0 (IBM Corp., Armonk, NY, USA), and a p-value <0.05 was considered statistically significant. The comparison between satisfied and non-satisfied patients was not pre-specified as a primary analysis; it was performed as an exploratory secondary subgroup analysis to examine whether subjective satisfaction was accompanied by differences in objective imaging parameters.

There were no missing data for the primary pre- and post-treatment ultrasonographic and elastographic measurements; therefore, no imputation was performed.

Sample size considerations

The sample size was determined based on feasibility considerations and consistency with previously published studies evaluating infraorbital rejuvenation using hyaluronic acid-based treatments. Given the exploratory single-arm interventional design and within-subject analytical approach, a formal a priori sample size calculation was not performed.

For paired measurements, a formal sample size estimation can theoretically be expressed using the following formula:

$n = \frac{(Z_{\alpha/2} + Z_{\beta})^{2} \times \sigma^{2}}{d^{2}}$

where n represents the required sample size, Zα/2 corresponds to the significance level, Zβ represents the desired statistical power, σ is the standard deviation of the paired differences, and d is the expected mean difference between pre- and post-treatment measurements [9]. In the present study, this formula was not applied to determine the final sample size because the required a priori assumptions, including the expected mean paired difference and the standard deviation of paired differences for the predefined ultrasonographic and elastographic outcomes, were not available before data collection. Therefore, the sample size should be interpreted as feasibility-based, and the findings should be considered preliminary and hypothesis-generating, particularly for secondary subgroup and treatment-response analyses.

Safety and patient satisfaction assessment

In terms of short-term safety, the treatment was well tolerated in the present cohort. No serious adverse events were observed during the three-month follow-up period, and all reported local reactions were mild, transient, and self-limited. The most common adverse effects included injection-site pain, bruising, and swelling. These findings are consistent with the expected safety profile of non-cross-linked hyaluronic acid-based formulations; however, the small sample size and short follow-up period should be considered when interpreting the safety findings.

The patient satisfaction assessment was conducted using a structured questionnaire developed by the authors specifically for this study. The questionnaire consisted of items evaluating overall satisfaction and perceived improvement in infraorbital appearance, including wrinkles, puffiness, hyperpigmentation, and skin quality. The full questionnaire is provided in Appendix A.

## Results

A total of 40 female patients were included in the study, and both infraorbital regions were treated, resulting in 80 treated eyelids. Demographic characteristics and patient-reported outcomes were interpreted at the patient level, whereas ultrasonographic, elastographic, and local safety outcomes were summarized per treated eyelid. The bilateral nature of the imaging data was considered when interpreting the findings. The mean age of the patients was 40.5 years, ranging from 23 to 53 years. Among the participants, 24 patients were smokers, and three patients had comorbidities, including Hashimoto’s thyroiditis, hypertension, and hypothyroidism. The average daily water consumption was 2 liters. In terms of patient satisfaction, 34 patients (85%) reported being satisfied with the procedure, while 6 patients (15%) were not satisfied. The mean physician-assessed procedural response score was 7 (Table 1).

**Table 1:** Baseline demographic and clinical characteristics of the study population This table presents the baseline demographic and clinical characteristics of the study participants. Continuous variables are expressed as mean ± standard deviation (SD), median, and minimum–maximum values, while categorical variables are presented as number (n) and percentage (%). Patient satisfaction and physician-assessed procedural response scores are also summarized.

Variable	Category	Min–Max	Median	Mean ± SD	n (%)
Age (years)	Overall	23.0–53.0	40.5	39.23 ± 8.39	—
Comorbidity	No	—	—	—	74 (92.5)
	Yes	—	—	—	6 (7.5)
	Hashimoto’s thyroiditis	—	—	—	2 (2.5)
	Hypertension	—	—	—	2 (2.5)
	Hypothyroidism	—	—	—	2 (2.5)
Smoking status	No	—	—	—	56 (70.0)
	Yes	—	—	—	24 (30.0)
Daily water consumption (liters)	Overall	0.5–3.0	2.0	1.84 ± 0.66	—
Nightly sleep amount (hours)	Overall	4.0–8.0	7.0	6.90 ± 1.31	—
Nightly sleep duration	4 hours	—	—	—	4 (5.0)
	5 hours	—	—	—	16 (20.0)
	7 hours	—	—	—	24 (30.0)
	8 hours	—	—	—	36 (45.0)
Patient satisfaction	No	—	—	—	12 (15.0)
	Yes	—	—	—	68 (85.0)
Procedure response score	Overall	1.0–9.0	7.0	6.28 ± 1.88	—
Lower eyelid epidermis–dermis thickness (mm)	Baseline	0.8–2.5	1.3	1.34 ± 0.34	—
Palpebral part of orbicularis oculi thickness (mm)	Baseline	0.4–1.6	0.9	0.95 ± 0.29	—
Lower eyelid epidermis–dermis elasticity (kPa)	Baseline	1.7–7.1	4.0	3.96 ± 0.95	—
Palpebral part of orbicularis oculi elasticity (kPa)	Baseline	2.2–6.8	4.2	4.39 ± 1.07	—
Retro-orbicularis oculi fat (ROOF) pad elasticity (kPa)	Baseline	2.1–7.7	4.2	4.44 ± 1.42	—

Lower eyelid epidermis-dermis thickness increased significantly from 1.34 ± 0.34 mm at baseline to 2.05 ± 0.39 mm after treatment, representing an approximately 53.0% increase (p < 0.001). Palpebral orbicularis oculi thickness also increased significantly from 0.95 ± 0.29 mm to 1.33 ± 0.41 mm, corresponding to an approximately 40.0% increase (p < 0.001).

Similarly, lower eyelid epidermis-dermis elasticity increased from 3.96 ± 0.95 kPa to 4.99 ± 1.28 kPa, representing an approximately 26.0% increase (p < 0.001), while palpebral orbicularis oculi elasticity increased from 4.39 ± 1.07 kPa to 5.36 ± 1.75 kPa, corresponding to an approximately 22.1% increase (p < 0.001). ROOF pad elasticity increased from 4.44 ± 1.42 kPa to 5.01 ± 1.77 kPa, corresponding to an approximately 12.8% increase; however, this change was not statistically significant (p = 0.068) (Table 2).

**Table 2:** Comparison of pre- and post-treatment ultrasonographic and elastographic parameters This table compares ultrasonographic and elastographic measurements obtained before and after treatment. Continuous variables are expressed as mean ± standard deviation (SD), median, and minimum–maximum values. Statistical comparisons between paired measurements were performed using the Wilcoxon signed-rank test. A p-value <0.05 was considered statistically significant.

		Min-Max			Median	Avg.±SD			p-value	
Lower eyelid epidermis-dermis thickness										
Before the procedure		0.8	-	2.5	1.3	1.34	±	0.34	<0.001	^w^
After the procedure		1.2	-	3.2	2.1	2.05	±	0.39		
Thickness of the palpebral part of the orbicularis oculi										
Before the procedure		0.4	-	1.6	0.9	0.95	±	0.29	<0.001	^w^
After the procedure		0.5	-	2.4	1.3	1.33	±	0.41		
Lower eyelid epidermis-dermis elasticity										
Before the procedure		1.7	-	7.1	4	3.96	±	0.95	<0.001	^w^
After the procedure		2	-	8.4	5	4.99	±	1.28		
Elasticity of the palpebral part of the orbicularis oculi										
Before the procedure		2.2	-	6.8	4.2	4.39	±	1.07	<0.001	^w^
After the procedure		2.4	-	10.5	5.1	5.36	±	1.75		
Retro-orbicularis oculi fat (ROOF) pad elasticity										
Before the procedure		2.1	-	7.7	4.2	4.44	±	1.42	0.068	^w^
After the procedure		2.1	-	9.8	4.6	5.01	±	1.77		
^w^ Wilcoxon test										

Satisfaction-based subgroup analyses were performed as exploratory secondary analyses. Because the non-satisfied subgroup was small, these comparisons were interpreted cautiously and were not considered confirmatory. Within-group pre- and post-treatment comparisons and between-group comparisons between satisfied and non-satisfied patients represent different statistical questions. Therefore, statistically significant within-group changes should not be interpreted as evidence of a significant difference in treatment effect between satisfaction groups unless the corresponding between-group comparison is also significant.

The lower eyelid epidermis-dermis thickness did not show a significant difference before and after the procedure between the satisfied and unsatisfied groups (p > 0.05). In the group unsatisfied with the procedure, the lower eyelid epidermis-dermis thickness showed a significant increase compared to before the procedure (p < 0.05). In the group who were satisfied with the procedure, the lower eyelid epidermis-dermis thickness also showed a significant elevation compared to before the procedure (p < 0.05).

The thickness of the palpebral part of the orbicularis oculi before and after the procedure showed no significant difference between the groups who were satisfied and not satisfied with the procedure (p > 0.05). The thickness of the palpebral part of the orbicularis oculi increased significantly compared to before the procedure in both groups that were unsatisfied with the procedure (p < 0.05).

There was no significant difference in the elastography of the eyelid epidermis-dermis before and after the procedure between the groups satisfied and unsatisfied with the procedure (p > 0.05). In both groups, satisfied and unsatisfied with the procedure, there was a significant increase (p < 0.05) in the elastography value of the lower eyelid epidermis-dermis compared to before the procedure.

In the group not satisfied with the procedure, there was no significant increase (p > 0.05) in the elastaography value of the palpebral part of the orbicularis oculi compared to before the procedure. However, in the group satisfied with the procedure, there was a significant increase (p < 0.05) in the elastography value of the palpebral part of the orbicularis oculi compared to before the procedure (Table 3).

**Table 3:** Comparison of demographic and clinical variables according to patient satisfaction. This table presents the comparison of demographic and clinical characteristics between patients who were satisfied and not satisfied with the treatment. Continuous variables are expressed as mean ± standard deviation (SD) and median values. The Mann–Whitney U test was used for comparisons of continuous variables, while categorical variables were analyzed using the chi-square test. A p-value <0.05 was considered statistically significant.

Variable	Category	Patient Satisfaction (-)		Patient Satisfaction (+)		p-value	Test
		Mean ± SD / n (%)	Median	Mean ± SD / n (%)	Median		
Age (years)	Overall	38.33 ± 8.39	41.50	39.38 ± 8.45	40.50	0.914	Mann-Whitney U
Comorbidity	No	10 (83.3)	—	64 (94.1)	—	0.191	Chi-square
	Yes	2 (16.7)	—	4 (5.9)	—	—	—
Comorbidity subtype	Hashimoto’s thyroiditis	2 (16.7)	—	0 (0.0)	—	—	—
	Hypertension	0 (0.0)	—	2 (2.9)	—	—	—
	Hypothyroidism	0 (0.0)	—	2 (2.9)	—	—	—
Smoking status	No	8 (66.7)	—	48 (70.6)	—	0.785	Chi-square
	Yes	4 (33.3)	—	20 (29.4)	—	—	—
Daily water consumption (liters)	Overall	1.50 ± 0.43	1.50	1.90 ± 0.68	2.00	0.034	Mann-Whitney U
Nightly sleep amount (hours)	Overall	6.00 ± 1.48	6.00	7.06 ± 1.22	7.50	0.011	Mann-Whitney U

Patients were classified into five categories based on their response to treatment: non-responsive, slightly responsive, moderately responsive, well-responsive, and very well-responsive.

When classified according to treatment response, there was no significant difference between the response groups regarding their age (p > 0.05). However, there was a significant difference between the response groups regarding the amount of water consumed daily (p < 0.05).

There was also a significant difference (p < 0.05) in the nightly sleep duration among the response groups. Additionally, there was a significant difference (p < 0.05) in the smoking status of patients among the response groups (with smoking showing a significant decrease in response) (Table 4).

**Table 4:** Distribution of clinical and ultrasonographic parameters according to treatment response categories. This table presents the distribution of clinical and ultrasonographic parameters across different treatment response categories (no response, slight response, moderate response, good response, and very good response). Continuous variables are expressed as mean values. Statistical significance was evaluated using appropriate non-parametric tests. A p-value <0.05 was considered statistically significant.

Variable		Response Groups					
		No Response	Slight Response	Moderate	Good Response	Very Good	p-value
		Mean	Mean	Mean	Mean	Mean	-
Age (years)		38	44	37	39	41	0.355
Lower eyelid epidermis-dermis thickness before the procedure (mm)		1.1	1.3	1.4	1.4	1.3	0.283
Lower eyelid epidermis-dermis thickness after the procedure (mm)		1.9	2.1	2	2.1	1.9	0.661
Thickness of the palpebral part of orbicularis oculi before the procedure (mm)		1	1	0.9	1	1	0.805
Thickness of the palpebral part of orbicularis oculi after the procedure (mm)		1.1	1.3	1.3	1.3	1.5	0.576
Lower eyelid epidermis-dermis elastography before the procedure (kPa)		3.6	3.8	4.2	4	3.3	0.076
Lower eyelid epidermis-dermis elastography after the procedure (kPa)		3.9	5.3	5.2	5	4.2	0.139
Mean elastography value of the palpebral part of orbicularis oculi before the procedure (kPa)		3.3	5	4.3	4.5	3.9	0.094
Mean elastography value of the palpebral part of orbicularis oculi after the procedure (kPa)		3.5	6	5.3	5.3	4.7	0.763
Retro-orbicularis oculi fat (ROOF) pad elastography before the procedure		6	5	4.4	4.5	4.1	0.599
ROOF pad elastography after the procedure		1.8	4.1	4.8	5.1	5.9	0.349
Daily water consumption (liters)		4.5	1.3	1.7	2	2.5	0.003
Sleep amount (hours)		-	6	6.5	7.5	7.7	<0.001
Smoking status	Yes	2	4	12	32	6	-
	No	2	6	8	8	0	-
Total		4	10	20	40	6	

Exploratory analyses were performed to evaluate whether baseline demographic and lifestyle characteristics were associated with patient satisfaction and physician-assessed treatment response. Age, comorbidity status, and smoking status did not differ significantly between satisfied and non-satisfied patients. However, daily water consumption and nightly sleep duration were higher among satisfied patients. When patients were categorized according to physician-assessed treatment response, age did not differ significantly across response groups, whereas daily water consumption, nightly sleep duration, and smoking status showed significant differences. Because these analyses were exploratory and the study was not designed to determine predictors of treatment response, these findings should be interpreted as associations rather than causal relationships.

Safety outcomes

No serious adverse events were observed during the three-month follow-up period. Mild and transient local reactions were reported in a small number of patients. Specifically, swelling was observed in five patients, resolving within 24 hours in three patients and persisting slightly longer in two patients. Bruising was reported in three treated eyelids. Mild injection-site pain or procedural discomfort was reported in all treated eyelids immediately after injection; however, it was self-limited, did not require additional treatment, and was not associated with any persistent pain or functional limitation. No systemic adverse effects or long-term complications were observed. All reported adverse events resolved spontaneously without the need for additional intervention (Table 5). Because safety monitoring was limited to the three-month follow-up period, delayed adverse events occurring beyond this period could not be assessed.

**Table 5:** Treatment-related adverse events per treated eyelids (n = 80) Values are presented as n (%). No serious adverse events were observed during the study period. All adverse events were mild, transient, and resolved spontaneously without the need for additional intervention.

Adverse event	Eyelids, n (%)	Clinical course
Swelling	5 (6.25)	Resolved within 24 hours in three eyelids; persisted slightly longer in two eyelids
Bruising	3 (3.75)	Mild; resolved spontaneously
Injection site pain	80 (100)	Mild and self-limited in all cases
Systemic adverse events	0 (0)	Not observed
Long-term complications	0 (0)	Not observed

## Discussion

The present study investigated short-term infraorbital tissue changes after non-cross-linked hyaluronic acid and amino acid-based treatment using quantitative B-mode ultrasonography and 2D-SWE. The main findings were significant increases in epidermis-dermis thickness, palpebral orbicularis oculi thickness, and selected elastographic parameters, whereas the increase in ROOF pad elasticity did not reach statistical significance. These findings suggest that the intervention was associated with measurable structural and biomechanical changes in superficial infraorbital tissues during the three-month follow-up period. However, because the study was conducted as a single-arm, open-label intervention without an untreated or placebo-controlled comparison group, these changes should be interpreted as preliminary within-subject observations rather than definitive evidence of treatment efficacy or causality.

Beyond statistical significance, the magnitude of the observed changes suggests that the treatment was associated with measurable tissue-level alterations. The approximately 53.0% increase in lower eyelid epidermis-dermis thickness and 40.0% increase in palpebral orbicularis oculi thickness indicate a substantial structural change on B-mode ultrasonography. Similarly, the approximately 26.0% increase in epidermis-dermis elasticity and 22.1% increase in palpebral orbicularis oculi elasticity suggest detectable biomechanical changes on 2D-SWE. Clinically, these changes may correspond to improved tissue hydration, dermal support, and perceived skin quality in the infraorbital region. However, clinical relevance should be interpreted cautiously because no validated aesthetic scale, minimal clinically important difference threshold, or blinded external assessment was used. Therefore, the observed imaging-based improvements should be considered objective evidence of short-term tissue change rather than definitive proof of clinically meaningful aesthetic improvement. Because formal effect size estimates and confidence intervals were not calculated, the magnitude and precision of these changes should be interpreted primarily based on the reported percentage changes and within the exploratory nature of the study.

The lack of statistical significance in ROOF elasticity despite an approximately 12.8% increase may be explained by several factors. First, the ROOF is a deeper and more heterogeneous fat-containing compartment compared with the epidermis-dermis layer and the palpebral portion of the orbicularis oculi muscle, which may increase measurement variability on 2D SWE. Second, the injected product was delivered into the deep dermis using a micropapular technique; therefore, the most direct tissue effect would be expected in more superficial layers rather than in the deeper ROOF compartment. Third, the relatively small sample size and short follow-up period may have limited the ability to detect smaller or more variable changes in ROOF elasticity. Accordingly, the non-significant ROOF finding should not be interpreted as definitive evidence of the absence of effect but rather as an inconclusive result requiring confirmation in larger controlled studies.

Infraorbital aging is a multifactorial process characterized by thinning of the dermis, loss of elasticity, redistribution of subcutaneous fat, and alterations in pigmentation. These structural and functional changes contribute to the clinical appearance of wrinkles, puffiness, and skin laxity [8,10]. Various non-surgical treatment modalities have been developed to address these concerns, among which hyaluronic acid-based therapies have gained widespread acceptance due to their biocompatibility and regenerative potential. The findings of the present study are consistent with previous reports demonstrating that hyaluronic acid-based treatments can improve dermal thickness, enhance elasticity, and contribute to overall skin quality [11-13]. However, direct comparison with previous studies should be made cautiously because of methodological differences. Earlier studies on hyaluronic acid-based rejuvenation have frequently focused on clinical appearance, patient satisfaction, or skin physiology in different anatomical regions, whereas the present study specifically evaluated the infraorbital region using quantitative B-mode ultrasonography and 2D-SWE. In addition, differences in product composition, injection technique, treatment intervals, follow-up duration, and outcome assessment tools may influence the comparability of results. Therefore, the present findings should be interpreted as complementary to previous clinical and physiological observations rather than directly equivalent to them.

Hyaluronic acid plays a central role in maintaining extracellular matrix integrity and tissue hydration [13]. Its interaction with CD44 receptors stimulates fibroblast proliferation and promotes collagen synthesis, particularly type III collagen, which is essential for dermal remodeling [14]. In addition, the amino acid components of the formulation contribute to collagen and elastin synthesis, thereby supporting tissue repair and improving structural integrity. These mechanisms may provide a biologically plausible explanation for the observed increases in tissue thickness and elasticity; however, they remain speculative in the context of the present study because molecular, biochemical, or histological validation was not performed.

The composition of Jalupro® Young Eye used in the present study is consistent with this rationale, as it combines non-cross-linked hyaluronic acid with amino acids that are structurally relevant to dermal extracellular matrix proteins. Therefore, the observed increases in epidermis-dermis thickness and elasticity may reflect, at least in part, hydration-related matrix effects and the supportive role of amino acid substrates in tissue remodeling. Nevertheless, this interpretation should be considered indirect, and the present imaging findings cannot confirm the underlying biological mechanisms.

The use of SWE in this study provided an objective and quantitative assessment of tissue biomechanical properties. Compared with conventional clinical evaluation methods, SWE offers improved reproducibility and reduced operator dependency, allowing for a more precise evaluation of treatment effects. The observed increase in elasticity values suggests that SWE may serve as a useful adjunctive quantitative imaging method for monitoring short-term infraorbital tissue changes; however, its measurement reliability was not formally validated in this cohort because intra-observer repeatability and inter-observer agreement analyses were not performed.

Notably, objective improvements were observed even in some patients who reported dissatisfaction with the treatment outcome. This discrepancy may reflect the fact that ultrasonographic and elastographic measurements quantify tissue-level changes, such as thickness and elasticity, whereas patient satisfaction is influenced by broader perceptual and aesthetic factors. In the infraorbital region, patients may judge treatment success based on visible changes in pigmentation, puffiness, wrinkles, symmetry, and overall facial appearance, which may not directly correlate with measurable changes in tissue thickness or stiffness. In addition, individual expectations, baseline aesthetic concerns, psychological perception of facial appearance, and the relatively subtle nature of early tissue-level changes may contribute to dissatisfaction despite objective improvement. Therefore, objective imaging findings and patient-reported satisfaction should be considered complementary rather than interchangeable outcome measures in aesthetic interventions. However, satisfaction-based subgroup findings should be interpreted cautiously because the non-satisfied subgroup was small and the analyses were exploratory, potentially limiting statistical reliability and increasing susceptibility to response and assessment bias.

Exploratory analyses also suggested that certain baseline lifestyle-related characteristics, including daily water consumption, nightly sleep duration, and smoking status, differed across satisfaction or treatment-response categories. These findings may indicate that general lifestyle and skin-related factors could influence perceived or physician-assessed response; however, they should be interpreted cautiously. The study was not powered or designed to identify independent predictors of treatment response, and potential confounding cannot be excluded.

In terms of short-term safety, the treatment was well tolerated in the present cohort. No serious adverse events were observed during the three-month follow-up period, and all reported local reactions were mild, transient, and self-limited. The most common adverse effects included injection-site pain or procedural discomfort, bruising, and swelling. These findings are consistent with the expected safety profile of non-cross-linked hyaluronic acid-based formulations; however, the small sample size and limited follow-up duration should be considered when interpreting the safety findings. Larger studies with longer follow-up are needed to more reliably assess uncommon or delayed adverse events.

Furthermore, although a subset of patients reported dissatisfaction, objective improvements in ultrasonographic and elastographic parameters were still observed in these individuals. Whether additional treatment sessions or longer follow-up periods could improve patient-perceived outcomes remains uncertain and should be evaluated in future controlled studies.

Limitations

This study has several limitations that should be acknowledged. Most importantly, the study was designed as a prospective, open-label, single-arm intervention without a parallel untreated or placebo-controlled group. Although the within-subject comparison allowed each participant to serve as her own baseline control and enabled quantitative assessment of pre- and post-treatment tissue changes, this design does not fully replace a controlled comparative design. Therefore, the observed changes cannot be attributed exclusively to the intervention, and potential confounding factors such as natural temporal variation, patient expectation, placebo-related effects, hydration status, and measurement-related variability cannot be completely excluded.

Satisfaction-based subgroup analyses were exploratory and limited by the small number of non-satisfied patients, which may reduce statistical reliability and increase susceptibility to response bias and assessment bias.

In addition, the relatively small sample size and the inclusion of only female participants may restrict the generalizability of the findings. Another limitation is that a formal a priori sample size calculation was not performed; therefore, the study may be underpowered for secondary subgroup analyses and for detecting smaller treatment-related differences. In addition, confidence intervals and formal effect size estimates were not reported, and no formal adjustment for multiple comparisons was applied. These factors may limit the precision of effect interpretation and increase the risk of type I error, particularly for secondary subgroups and treatment-response analyses. The follow-up period was limited to three months, which may not fully capture long-term durability. Although objective ultrasonographic and elastographic measurements were used, subjective patient-reported outcomes may have been influenced by individual expectations and perception bias. Furthermore, the 10-point physician response scale and author-developed satisfaction questionnaire were not validated aesthetic outcome instruments, and validated scales such as the GAIS were not used. Because the satisfaction questionnaire was developed specifically for this study, direct comparison of patient-reported outcomes with other studies using validated or different aesthetic assessment tools is limited. In addition, clinical outcome assessments were not blinded, which may have introduced assessment bias, particularly for physician-assessed responses and patient-reported outcomes. Therefore, the subjective clinical outcomes should be interpreted with caution and considered complementary to the imaging-based quantitative findings. Finally, the lack of randomization may introduce potential selection bias. Accordingly, the results should be interpreted primarily in the context of female patients, and future studies should include male participants to determine whether comparable ultrasonographic and elastographic tissue responses are observed across sexes. Although a standardized imaging protocol was used and three repeated measurements were averaged for each anatomical region, formal intra-observer repeatability and inter-observer agreement analyses were not performed. Therefore, potential measurement variability related to observer dependency cannot be fully excluded, representing an important limitation for imaging-based outcomes and for the interpretation of treatment-related changes in tissue thickness and elasticity.

Another methodological limitation is that bilateral infraorbital measurements were summarized per treated eyelid, although two treated eyelids belonged to the same patient. Therefore, potential within-patient correlation between bilateral measurements cannot be completely excluded, and this should be considered when interpreting the imaging-based findings.

Future investigations should include an untreated, placebo, or active-comparator parallel control group to validate whether the observed ultrasonographic and elastographic changes exceed nonspecific temporal variation or expectation-related effects.

## Conclusions

In conclusion, two sessions of non-cross-linked hyaluronic acid combined with a defined amino acid complex, administered at three-week intervals, were associated with measurable structural and elastographic changes in the infraorbital region. Specifically, the treatment was accompanied by increases in epidermis-dermis thickness, palpebral orbicularis oculi thickness, and tissue elasticity, as demonstrated by B-mode ultrasonography and 2D-SWE.

Notably, although a subset of patients did not report subjective satisfaction, objective improvements were still observed, highlighting the importance of combining patient-reported outcomes with quantitative imaging modalities in the evaluation of aesthetic interventions. In the present cohort, this minimally invasive treatment approach was associated with mild clinical improvement supported by quantitative imaging findings. However, further studies with larger sample sizes, longer follow-up durations, and controlled study designs are warranted to validate and expand upon these findings.

## Appendices

Appendix A

Patient Satisfaction Questionnaire

1. Are you satisfied with the overall outcome of the treatment? (Yes/No)

2. Did you observe an improvement in the appearance of wrinkles in the infraorbital area? (Yes/No)

3. Did you observe a reduction in puffiness around the eyes? (Yes/No)

4. Did you observe an improvement in skin quality (e.g., smoothness, hydration)? (Yes/No)

5. Did you observe any improvement in hyperpigmentation in the infraorbital region? (Yes/No)

## References

[1] Bravo BS, Rocha CR, Bastos JT, Silva PM (2015). Comprehensive treatment of periorbital region with hyaluronic acid. J Clin Aesthet Dermatol.

[2] Kołodziejczak A, Rotsztejn H (2022). The eye area as the most difficult area of activity for esthetic treatment. J Dermatolog Treat.

[3] Glaser DA, Kurta A (2016). Periorbital rejuvenation: overview of nonsurgical treatment options. Facial Plast Surg Clin North Am.

[4] Aşiran Serdar Z, Altan Ferhatoğlu Z (2021). Mesotherapy and platelet rich plasma applications in the periorbital region (Article in Turkish). Türkiye Klinikleri.

[5] Muiznieks LD, Keeley FW (2013). Molecular assembly and mechanical properties of the extracellular matrix: a fibrous protein perspective. Biochim Biophys Acta.

[6] Sugahara KN, Murai T, Nishinakamura H, Kawashima H, Saya H, Miyasaka M (2003). Hyaluronan oligosaccharides induce CD44 cleavage and promote cell migration in CD44-expressing tumor cells. J Biol Chem.

[7] Poleva I (2022). Perioral rejuvenation: evaluation of safety and efficacy of high molecular weight hyaluronic acid and amino acid treatment. J Drugs Dermatol.

[8] Swift A, Liew S, Weinkle S, Garcia JK, Silberberg MB (2021). The facial aging process from the “inside out”. Aesthet Surg J.

[9] Chow S-C, Shao J, Wang H (2008). Sample Size Calculations in Clinical Research. Chapman & Hall/CRC.

[10] Langton AK, Sherratt MJ, Griffiths CE, Watson RE (2010). A new wrinkle on old skin: the role of elastic fibres in skin ageing. Int J Cosmet Sci.

[11] Solano F (2020). Metabolism and functions of amino acids in the skin. Adv Exp Med Biol.

[12] Williams S, Tamburic S, Stensvik H, Weber M (2009). Changes in skin physiology and clinical appearance after microdroplet placement of hyaluronic acid in aging hands. J Cosmet Dermatol.

[13] Matarasso SL (2004). Understanding and using hyaluronic acid. Aesthet Surg J.

[14] Doillon CJ, Silver FH, Berg RA (1987). Fibroblast growth on a porous collagen sponge containing hyaluronic acid and fibronectin. Biomaterials.

